# Recurrent Reactive Infectious Mucocutaneous Eruption in an Adult Male Secondary to Mycoplasma Infection: A Case Report

**DOI:** 10.7759/cureus.60603

**Published:** 2024-05-19

**Authors:** Laxman Wagle, Parmartha Basnyat, Anuj Timshina, Rashmita Regmi, Rushika Ban

**Affiliations:** 1 Internal Medicine, Ascension Saint Agnes Hospital, Baltimore, USA; 2 Internal Medicine, Patan Academy of Health Science, Kathmandu, NPL; 3 Nursing, Karnali Academy of Health Science, Jumla, NPL; 4 Pediatric Medicine, Sinai Hospital of Baltimore, Baltimore, USA

**Keywords:** systemic steroid therapy, oral ulcer, mycoplasma pneumonia-induced rash and mucositis, reactive infectious mucocutaneous eruptions, mycoplasma

## Abstract

Mycoplasma pneumoniae commonly causes respiratory tract infections but can also involve the skin and mucosal surfaces. Reactive infectious mucocutaneous eruption (RIME) secondary to mycoplasma infection is uncommon in adults but is an important clinical entity. We present the case of a 26-year-old male who experienced recurrent episodes of erythematous and painful oral ulcers without any prodromal or respiratory symptoms. Serological testing confirmed a recent mycoplasma infection. The patient was successfully treated with oral steroids and supportive therapy. This case underscores the challenges of diagnosing RIME, particularly in the absence of typical respiratory symptoms. Moreover, oral steroid therapy with supportive treatment may suffice to manage RIME if the patient lacks an ongoing infection or other underlying pathologies.

## Introduction

Mycoplasma pneumoniae is an atypical bacterium without a cell wall that commonly causes respiratory tract infections in adult and pediatric populations. It is also a leading cause of community-acquired pneumonia and can cause respiratory symptoms and extrapulmonary diseases in up to 25% of patients [1,2]. Although rare, Mycoplasma infection can cause a variety of non-respiratory presentations with or without respiratory tract involvement and stem from direct invasion or an autoimmune response. It can cause hemolytic anemia, rashes, encephalitis, meningitis, pericarditis, endocarditis, hepatitis, and several other organ-system involvements [2,3]. Cutaneous manifestations commonly include mucocutaneous eruptions resembling Stevens-Johnson syndrome (SJS), previously called Mycoplasma pneumonia-induced rash and mucositis (MIRM), and are now categorized under the broad term called reactive infectious mucocutaneous eruption (RIME) to differentiate it from non-infectious etiologies, including drugs [4].

RIME is a clinical entity encompassing a post-infectious rash and mucositis and is widely observed in young patients. It has been reported to be caused by various respiratory infections, including viral infections; however, the most common cause has been described as M. pneumoniae. Although it primarily occurs as a vesiculobullous lesion in the oral, ocular, and genital mucosa, most patients have oral mucosal involvement (up to 100%) and can recur [4,5].

## Case presentation

**Figure 1:**
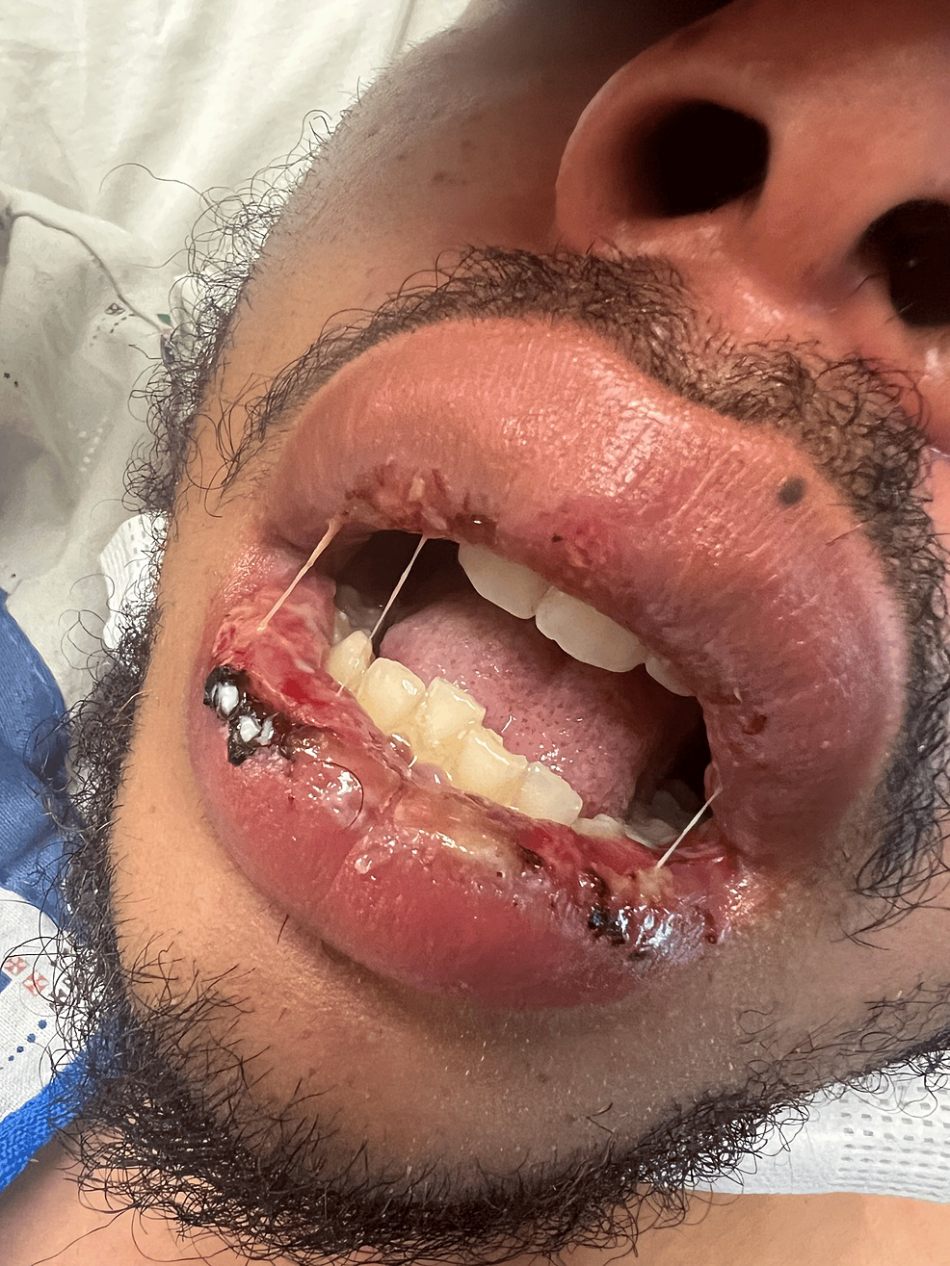
Lip lesion on the first day

**Figure 2:**
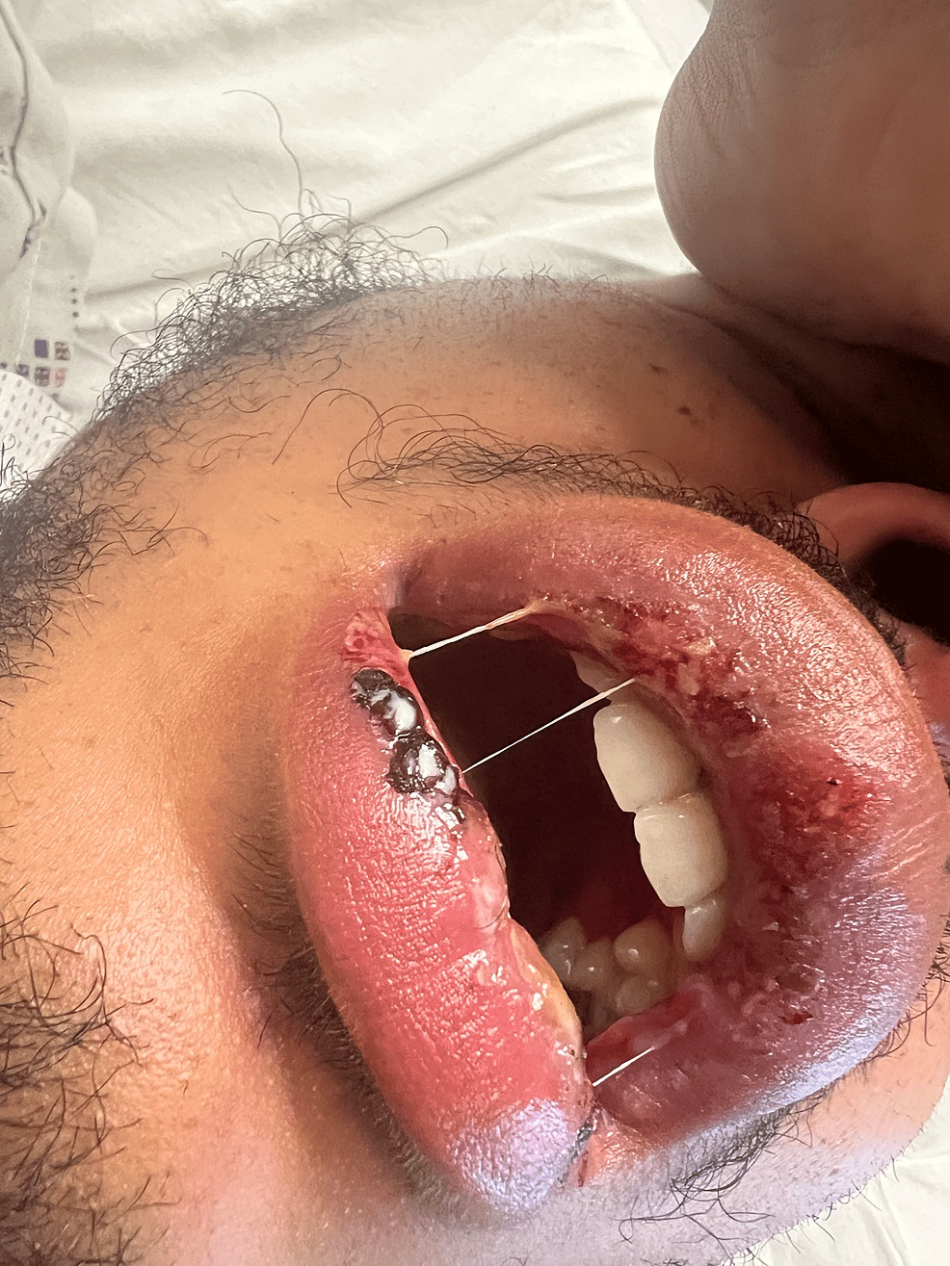
Lip lesions on the second day

We report the case of a 26-year-old male with a history of recurrent oral ulcers since the age of 19 years who presented to the emergency department with severe throat pain, fever, and odynophagia for two days. He experienced sudden and severe throat pain, along with a feeling of throat closure. He had lesions on his lips (Figures 1-2) and mouth. He denied having rashes anywhere else on the body. He denied any family history of a similar rash, autoimmune diseases, recent sick contact, or travel. He is monogamous in a relationship.

He had an oral ulcer with minimal lip involvement without other symptoms that required an emergency visit one year before presentation. He had extensive workups performed at that time, including serological tests for HIV, syphilis, gonorrhea, chlamydia, COVID-19, influenza, rapid streptococcal test, herpes simplex virus polymerase chain reaction (PCR), and even biopsy with PCR negative for HSV and varicella-zoster virus (VZV), all of which were all negative. Mycoplasma serology was performed at that time and showed IgG levels of 500 U/mL (units/mL) (reference value <100 U/mL) and IgM levels of 1,115 U/mL (reference value <770 U/mL). He was given oral valacyclovir for 14 days, oral dexamethasone swish and spit, and a mouthwash containing lidocaine. His symptoms improved; however, he was lost to outpatient follow-up with dermatology.

On admission, the patient had a temperature of 38.1°C, heart rate of 108 beats per minute, blood pressure of 120/69 mmHg, and oxygen saturation of 97% on room air. Physical examination showed shallow ulcers on the lips (Figures 1-2) and vesiculobullous coalescing ulcers with surrounding erythema in the hard palate, soft palate, and oro-pharynx. The initial laboratory findings revealed neutrophilic leukocytosis of 15,000 (neutrophils of 75.4%, lymphocytes of 9.1%, monocytes of 14.5%, eosinophils of 0.1%, and basophils of 0.3%), and the basic metabolic profile was unremarkable. The rapid group A strep test results were negative. Throat culture showed normal respiratory flora. Computed tomography (CT) of the neck showed bilateral reactive lymphadenopathy without evidence of mass, abscess, or drainable fluid collections. Chest x-ray was normal. Mycoplasma IgG titers were greater than 891 U/mL (reference value <100 U/mL), and mycoplasma IgM titers were greater than 1,335 U/mL (reference value <770 U/mL). However, respiratory viral panels and HSV PCR returned negative results, leading to a diagnosis of RIME secondary to mycoplasma infection (Table 1).

**Table 1:** Respiratory viral panel

Respiratory Viruses	Results
Nasal Adenovirus PCR	Not detected
Nasal B. parapertussis DNA PCR	Not detected
Nasal Coronavirus 229E PCR	Not detected
Nasal Coronavirus HKU1 PCR	Not detected
Nasal Coronavirus NL63 PCR	Not detected
Nasal Coronavirus OC43 PCR	Not detected
Nasal Enterovirus/Rhinovirus PCR	Not detected
Nasal Influenza A PCR	Not detected
Nasal Influenza A H1 PCR	Not detected
Nasal Influenza A H1 2009 PCR	Not detected
Nasal Influenza A H3 PCR	Not detected
Nasal Influenza B PCR	Not detected
Nasal Parainfluenza 1/2/3/4 PCR	Not detected
Nasal RSV PCR	Not detected
Nasal B. pertussis DNA PCR	Not detected
Nasal C. pneumoniae PCR	Not detected
Nasal Human Metapneumo PCR	Not detected
Nasal SARS-CoV-2 PCR	Not detected

**Figure 3:**
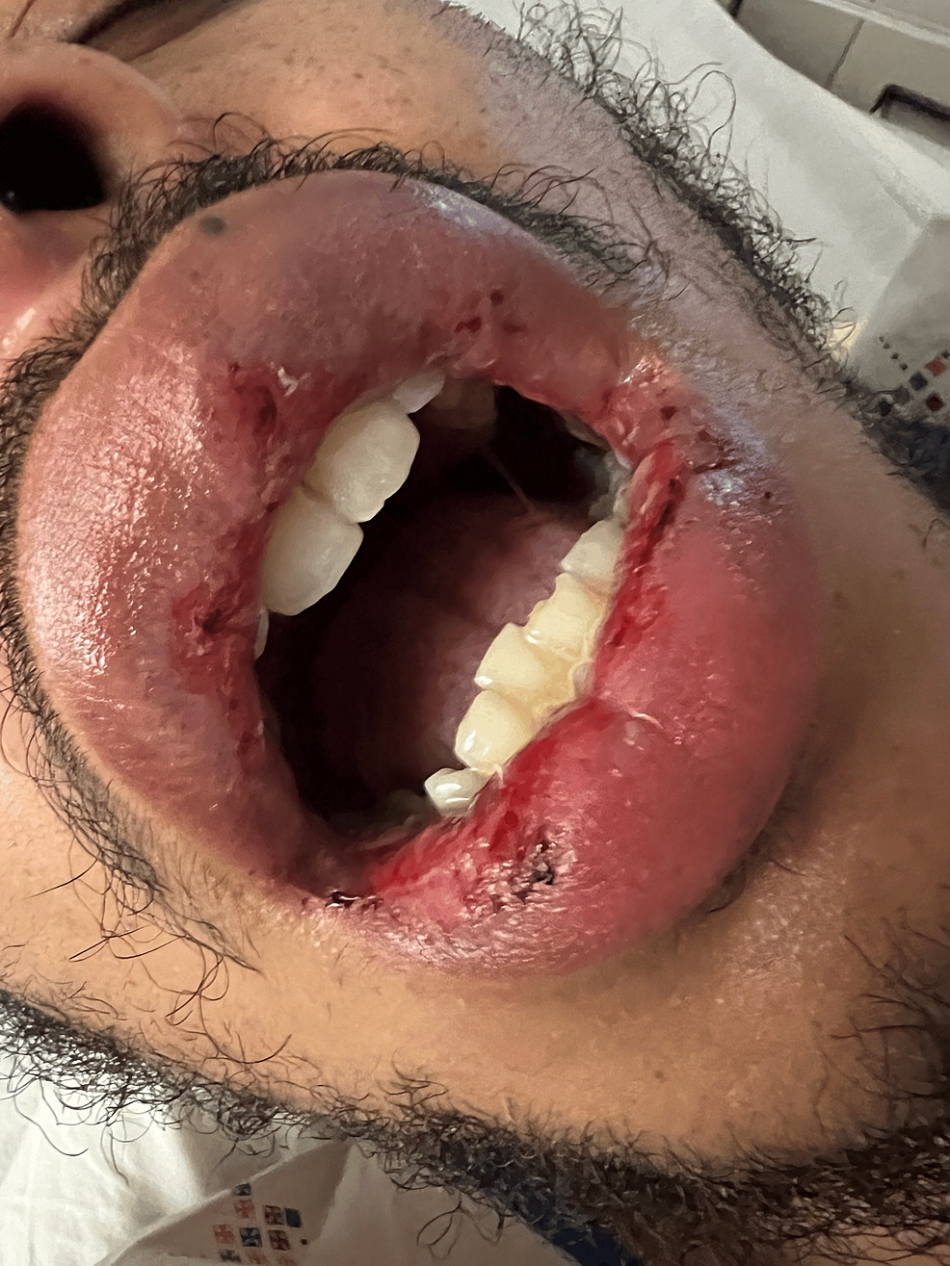
Lip lesions after starting oral steroid on the third day

Due to the extensive mucositis with oral ulcers, the patient was initially treated with IV steroids and valacyclovir until the result of HSV PCR was obtained, which was negative. The patient’s symptoms worsened on the second day of treatment. Oral dexamethasone swish and spit therapy was started, where the patient would swish and spit out a solution containing the corticosteroid dexamethasone. A mouthwash containing the local anesthetic lidocaine was continued. Valacyclovir and IV steroids were discontinued. His symptoms began to improve with the improvement of the ulcers on his lips (Figure 3) and oral ulcers. The patient was able to consume a liquid diet; subsequently, a soft diet was initiated on the fourth day. His lips and oral lesions resolved within two weeks. He was advised to follow up with the dermatology department on an outpatient basis.

## Discussion

RIME, previously called M. pneumonia-induced rash, describes mucocutaneous eruptions in patients with M. pneumoniae infections. Other terms, such as SJS, erythema multiforme, mucositis, and Fuchs syndrome, have also been used [4]. Most of the cases of RIME are seen in young patients, with an average age between 5.7 and 12 years, male predominance, and can occur in up to 25% of patients with M. pneumoniae infection [4-6]. Only a few cases of RIME in adults have been reported and attributed to various infectious causes, including COVID-19 infection, M. pneumoniae, and Chlamydophila pneumoniae, making RIME a relatively novel diagnosis for adults [7-9].

RIME usually manifests as vesiculobullous or targetoid lesions on the oral mucosa. It can also be an erosion, ulcer, papules, macules, or morbilliform eruption. While the oral mucosa is involved in almost all cases (96-100%), ocular, urogenital, and cutaneous lesions can also be present [4-6,10]. Canavan et al. [4] analyzed 202 pediatric cases of M. pneumonia-associated mucocutaneous disease and noted that very few had cutaneous involvement, with recurrence occurring in 8% of the cases. Although Mycoplasma infections commonly involve the respiratory tract, extrapulmonary complications can occur independently or before the manifestation of respiratory symptoms. Owing to the complexity and varied presentation of Mycoplasma, it may be difficult to include it in the list of possible diagnoses. A retrospective cohort study conducted at Boston Children’s Hospital identified that recurrent cases of RIME were less likely to have infectious prodrome or non-oral mucosal involvement, as in our case [5].

RIME, HSV-related Erythema multiforme, and drug-induced SJS/TEN share similar mucocutaneous features. It can sometimes be challenging to differentiate between the three without a proper history and if distinguishing features are absent. Our 26-year-old male patient also had recurrent vesiculobullous lesions in the oral mucosa with no ocular or skin involvement, making it necessary to consider other differential diagnoses. Similar to other diseases, RIME can also lead to severe mucosal complications. Serology, including M. pneumoniae IgM antibodies or PCR, can aid in the laboratory diagnosis of the causative organism.

Although treatments for RIME have not been established, several studies have shown that antibiotics, systemic and topical corticosteroids, supportive care, and intravenous immunoglobulin (IVIG) can be used [4,5]. Recent studies of immunomodulators such as etanercept and cyclosporine have also demonstrated their effectiveness [11,12]. Etanercept has shown better outcomes than other treatment options, including supportive care, cyclosporine, IVIG, and systemic corticosteroids [11]. Furthermore, corticosteroid therapy and supportive treatment have shown better results than IVIG, suggesting that IVIG may not be effective in its management [13]. Initially, the patient was managed with supportive therapy and antibiotics (doxycycline), which were discontinued after normal radiographic findings. Antiviral treatment was also initiated but subsequently halted upon negative PCR results for HSV. Ultimately, the patient's condition improved with topical corticosteroids and supportive therapy.

## Conclusions

Mucocutaneous lesions, as seen in conditions such as MIRM/RIME, should raise suspicion for a Mycoplasma infection, even when typical respiratory symptoms are absent. In cases in which patients lack respiratory symptoms and chest findings, antibiotic therapy may not be deemed necessary, as the extrapulmonary manifestations of MIRM may stem from an autoimmune or inflammatory response rather than a direct infectious cause. In such instances, oral steroids (dexamethasone swish and spit), along with supportive therapy, could be sufficient for management.
